# Association of serum methylglyoxal with endothelial dysfunction in patients with type 2 diabetes

**DOI:** 10.3389/fphar.2026.1773149

**Published:** 2026-02-04

**Authors:** Shanshan Yu, Xinyan Jin, Yuanying Xu, Zhao Liu, Jun Lu, Jie Gao, Wenjun Sha, Tao Lei

**Affiliations:** Department of Endocrinology, Putuo Hospital, Shanghai University of Traditional Chinese Medicine, Shanghai, China

**Keywords:** association, biomarker, diabetes mellitus, endothelial dysfunction, methylglyoxal

## Abstract

**Aims:**

To explore the correlation between serum Methylglyoxal (MGO) and endothelial dysfunction in patients with type 2 diabetes mellitus, and to evaluate the clinical value of MGO in the development of diabetes mellitus and its complications.

**Methods:**

In this cross-sectional study, we enrolled 250 patients diagnosed with T2MD. Based on flow-mediated dilation (FMD) measurements, the patients were categorized into normal endothelial function group (FMD ≥6.4%, n = 61) and endothelial dysfunction group (FMD <6.4%, n = 189). Analysis of the relationship between MGO and FMD was conducted via Spearman’s correlation, partial correlation, and multiple logistic regression. An ROC curve analysis was utilized to quantify the predictive performance of MGO for endothelial function.

**Results:**

Endothelial dysfunction was observed in 189 (76%) patients with type 2 diabetes. Patients with endothelial dysfunction had higher concentration of MGO in the serum (P < 0.001) than those without endothelial dysfunction. Spearman correlation analysis showed that there was a significantly negative correlation between FMD and MGO (R = −0.611, *p* < 0.001), and this negative correlation remained significant upon adjustment for age and sex. (R = −0.36, *p* < 0.001). Logistic regression analysis identified MGO as an independent risk factor for endothelial dysfunction (OR 1.099, (1.06–1.14), p < 0.001), and the odds of endothelial dysfunction increased 2.67-fold per standard deviation (SD) increment in MGO levels (OR: 2.67 (1.78–4.01), *p* < 0.001) (Model 1). After adjusting for gender, age, BMI, course of disease, hypertension, smoking and alcohol consumption (model 2) as well as HbA1c, HOMA-IR, C-reactive protein and TG (model 3), similar results were obtained. Restricted cubic spline (RCS) analysis revealed a significant non-linear does-response relationship between MGO levels and endothelial function (*P*_overall_<0.001, *P*_non-linearing_<0.001). Subgroup analyses demonstrated that the association between MGO levels and endothelial function remained consistent across various strata, including age, sex, and comoribidities (all *P*_interaction_>0.05). Receiver operating characteristic (ROC) curve: the area of under the ROC curve (AUC) for MGO was 0.785 (OR: 0.73-0.84, p < 0.001).

**Conclusion:**

MGO was significantly inversely associated with FMD and endothelial function in T2DM patients, and can be used as a biomarker to assess vascular endothelial health. Detection of serum MGO levels has clinical significance in the prevention of early diabetic vascular disease.

## 1 Introduction

Diabetes mellitus (DM) is a chronic metabolic disease, which is caused by the defect of islet function secretion or the impairment of other biological functions (Tufail et al., 2025). According to the data of the International Diabetes Federation (IDF) in 2025, the number of diabetes patients aged 20 to 79 in the world is as high as 589 million, and is expected to increase to 853 million by 2050, accounting for 13% of the global population.In 2024, more than 3.4 million people died of diabetes and its complications worldwide, accounting for 9.3% of the total number of deaths worldwide (Cai et al., 2024). Diabetes mellitus has emerged as a major global public health challenge that poses a serious threat to human health. In addition, the total healthcare expenditure related to diabetes and its complications in the world exceeded $1 trillion for the first time in 2024, posing a serious challenge to the global health system (International Diabetes Federation, 2025).

Type 2 diabetes is characterized by multiple metabolic disorders characterized by chronic glucose homeostasis imbalance, increased oxidative conditions, and disturbances in carbohydrate, fat, and protein metabolism (Twarda-Clapa et al., 2022). Methyl glyoxal (MGO) is an α-oxaldehyde, a highly reactive acyclic dicarbonyl compound produced during the oxidation of sugars, and a by-product of glucose, lipid, and amino acid metabolism (Abidi et al., 2018). They react readily with lysine and arginine residues of lipids, nucleic acids, and proteins to form advanced glycation end products (Advanced Glycated Endproducts, AGEs); Hyperglycemia, decreased glucose utilization, and phosphotriose isomerase deficiency lead to increased plasma MGO levels and decreased levels of the reducing product glutathione, which together cause oxidative stress and damage vascular endothelial function, thereby inducing a variety of complications, mainly vascular disease (Vašková et al., 2025).

Vascular complications of diabetes involve heart, brain, kidney, retina and other important organs, which is the leading cause of death and disability in diabetic patients (Sallam and Laher, 2020). Clinical studies have shown that the age of atherosclerosis in diabetic patients is younger, the severity is higher, and the scope of lesions is wider. Atherosclerosis is the core manifestation of diabetic vascular lesions, and endothelial dysfunction is the initiating factor of atherosclerosis. The three affect each other (Lee et al., 2023). As a highly efficient glycation agent with 20,000 times higher reactivity than glucose, MGO can induce the growth of necrotic core of plaque in diabetic atherosclerosis patients, which makes the plaque prone to rupture and accelerates the development of diabetic complications (Schalkwijk et al., 2023; Hanssen et al., 2018).

Therefore, based on the universality and harmfulness of diabetic vascular complications, it is very important to evaluate and prevent the early vascular injury of diabetes. The assessment of endothelial function is based on the traditional invasive percutaneous quantitative coronary angiography, but it has limitations in clinical screening (Baltu et al., 2019). Flow-mediated dilation (FMD) of the brachial artery is a new noninvasive technique for assessing vascular endothelial function.In terms of assessment results, FMD is highly correlated with the gold standard quantitative coronary angiography, and has become a good indicator for replacing coronary angiography (Flammer et al., 2012). Studies have shown that a 1% reduction in FMD increases the risk of cardiovascular events by 13% (Hashimoto et al., 2021; Welters et al., 2017).

Vascular disease caused by diabetes mellitus involves heart, brain and other important organs, which is the main cause of death and disability in diabetic patients (Khan and Jandeleit-Dahm, 2025). Related studies have shown that vascular endothelial damage caused by endothelial dysfunction is an important factor in vascular disease (Pasut et al., 2025; Ji et al., 2025). MGO promotes endothelial dysfunction through oxidative stress and inflammation (Wan et al., 2024). Therefore, elucidating the relationship between MGO and endothelial dysfunction holds important clinical implications for preventing diabetic vascular complications.This study was to investigate the correlation between MGO and endothelial dysfunction in type 2 diabetes mellitus, to evaluate the clinical value of MGO in diabetes mellitus and its complications, and to provide a basis for early clinical diagnosis of diabetic vascular lesions.

## 2 Materials and methods

### 2.1 Object of study

In this cross-sectional study, 273 patients diagnosed with T2MD were recruited in the Department of Endocrinology, Putuo Hospital, Shanghai University of Traditonal Chinese Medicine from 1 January 2024 to 22 November 2024. The diagnostic criteria were in line with the diagnostic criteria of diabetes mellitus issued by WHO in 1999. Exclusion criteria: patients with diabetic ketoacidosis; patients with severe hepatic and renal dysfunction; patients with malignant tumors; patients with severe anemia, infection or trauma, and autoimmune diseases; patients during pregnancy and lactation; and patients with cognitive dysfunction or psychiatric disorders.

In order to achieve the aim of research, we excluded patients with following conditions: (1) diabetic ketoacidosis (n = 5), (2) severely liver and kidney dysfunction (n = 8) (3) and patients with malignant tumors (n = 10). Ultimately, 250 patients were collected for this ivestiggation. Finally, 250 patients were enrolled. According to the results of FMD, the patients were divided into normal endothelial function group (FMD ≥6.4%, n = 61) and endothelial dysfunction group (FMD <6.4%, n = 189).

Ethical approval for this study was granted by the Institutional Review Board of Putuo Hospital, Shanghai University of Traditional Chinese Medicine. All procedures were performed in full compliance with the ethical standards of the revised Helsinki Declaration. Written informed consent was obtained from all individual participants prior to any study procedures, and all personally identifiable information was held in strict confidence.

### 2.2 Anthropometric and laboratory measurement

Diagnostic criteria for type 2 diabetes: (1) typical diabetic symptoms (polydipsia, polyuria, polyphagia, unexplained weight loss) plus random blood glucose ≥11.1 mmol/L; (2) Fasting blood glucose ≥7.0 mmol/L or blood glucose ≥11.1 mmol/L 2 h after glucose loading (3) Glycosylated hemoglobin ≥6.5% (4) Patients without typical symptoms of diabetes should be reexamined another day for confirmation.

The general clinical data and laboratory biochemical indicators of the patients in the two groups were collected, and the concentration of MGO in the serum of the patients was measured. Clinical information including age, gender, height, weight, course of diabetes, smoking history, drinking history, history of hypertension were collected and BMI was calculated. After fasting for 12 h, 3–5 mL venous blood was drawn on an empty stomach the next morning, and glycosylated hemoglobin, FPG, TC, TG, HDL-C, LDL-C and fasting insulin were detected by Roche cobas8000 automatic biochemical analyzer. The HOMA-IR calculation formula was (fasting blood glucose * fasting insulin)/22.5. Glycosylated hemoglobin (HbA1c) was detected by BC -5390 glycosylated hemoglobin analyzer (Premier Hb9210) and high performance liquid chromatography.

Measurement of FMD: Subjects were instructed to fast for at least 4 h before the test and to abstain from smoking, alcohol, caffeine, or antioxidant vitamins for at least 12 h before the test.Patients were asked to rest in a sitting position in a quiet, dark, air-conditioned room (22 °C–25 °C) for at least 5 min before their blood pressure was measured by sphygmomanometry (Yoshida et al., 2010). FMD measurements were then performed after the patient was in the same room, resting in the prone position for at least 15 min. Ultrasound measurements were performed according to the guidelines for ultrasound assessment of brachial artery FMD. Calculation formula: FMD (%) = [maximum vessel diameter (mm)-vessel diameter at rest (mm)]/vessel diameter at rest (mm) * 100%.

After the whole blood sample was collected and kept at room temperature for 2 h, it was centrifuged for 15 min with a centrifuge at a speed of 2000 rpm. The supernatant of the blood sample was taken and packed into 1.5  mL EP tubes and stored in a refrigerator at a low temperature of −80 °C. Serum MGO concentrations were measured using a human MGO enzyme-linked immunosorbent assay (ELISA) kit (Fusheng Industrial Co., Ltd., China), all in strict accordance with the instructions.

### 2.3 Statistical methods

Statistical analyses were conducted using SPSS for Windows version 27.0. The Kolmogorov–Smirnov test evaluated the distribution of all continuous variables; those with a normal distribution were presented as mean ± standard deviation (®*x* ± *s*). Continuous variables that did not follow a normal distribution were reported as median (lower quartile, upper quartile) [M (QL, QU)], while categorical variables were expressed as counts and percentages (n [%]). Group differences were assessed using the Mann–Whitney U test for continuous variables and the chi-square test for categorical variables. Spearman’s correlation and multivariable logistic regression analyses were performed to examine the relationship between FMD and MGO. Additionally, the ROC curve was employed to evaluate the sensitivity and specificity of MGO in predicting endothelial dysfunction.

## 3 Results

### 3.1 Clinical data characteristics of patients from both groups were included

Table 1 presents the fundamental clinical characteristics of the patients who were ultimately enrolled. This study included a total of 189 patients diagnosed with endothelial dysfunction. The individuals in the endothelial dysfunction group were clearly older than those in the group without endothelial dysfunction. (67 vs. 63, *p* < 0.001). Patients with endothelial dysfunction had significantly higher MGO levels than those without endothelial dysfunction (289.56 (236.44, 326.81) vs. 216.68 (199.32, 235.83), *p* < 0.001). No significant differences were observed between the study groups regarding gender, smoking status, alcohol consumption, and disease duration.

**TABLE 1:** Clinical characteristics of patients included.

Variables	All (n = 250)	Nonendothelial dysfunction (n = 61)	Endothelial dysfunction (n = 189)	p for trend
Gender, male	151 (60.4)	31 (50.8)	120 (63.5)	0.078
Age, years	66.0 (60, 71)	63.0 (54.0, 70.0)	67.0 (60.5, 71.0)	<0.001
Duration of diabetes, years	10.0 (3, 20)	6.0 (2.5, 15.0)	10.0 (4, 20.0)	<0.001
Current smoker, %	46 (18.4)	12 (19.7)	34 (18)	0.768
Current drinkers, %	43 (17.2)	9 (14.8)	34 (18)	0.560
BMI, kg/m^2^	24.2 (22.5, 26.7)	23.9 (21.8, 25.8)	24.4 (22.6, 26.9)	<0.001
SBP, mmHg	130 (125, 140)	130 (124, 139)	130 (125, 140)	<0.001
DBP, mmHg	80 (77, 88)	80 (79, 90)	80 (77, 88)	<0.001
Hypertension (%)	155 (62)	29 (47.5)	126 (66.7)	0.007
FPG, mmol/L	7.13 (5.78,10.06)	6.84 (5.91, 10.68)	7.20 (5.63, 9.85)	<0.001
HbA1c, %	9.3 (7.9,11.1)	9.5 (7.9–11.5)	9.2 (7.6–10.9)	<0.001
FCP, pmol/L	0.7 (0.43,1.04)	0.67 (0.47, 0.93)	0.71 (0.42, 1.04)	<0.001
F-INS, pmol/L	74.39 (50.75, 132.6)	72.5 (49.39,157.5)	74.97 (50.84,125.7)	<0.001
HOMA-IR, mmol/L*mU/L	25.66 (15.26, 50.4)	25.97 (15.25,60.3)	25.47 (15.06,47.61)	<0.001
TC, mmol/L	4.41 (3.55, 5.36)	4.80 ± 1.35	4.34 (3.53, 5.28)	<0.001
TG, mmol/L	1.41 (1.06, 2.21)	1.31 (0.97, 1.79)	1.44 (1.06, 2.30)	<0.001
HDL-C, mmol/L	1.08 (0.92, 1.32)	1.14 ± 0.27	1.11 (0.82, 1.34)	<0.001
LDL-C, mmol/L	2.61 (1.93, 3.5)	3.02 ± 1.15	2.49 (1.89, 3.28)	<0.001
MGO, Pg/ML	269.66 (214.00, 314.81)	216.68 (199.32, 235.83)	289.56 (236.44, 326.81)	<0.001
FMD (%)	5.3 (3.78,6.3)	7.4 (6.85, 8.55)	4.7 (3.2, 5.7)	0.033

Data were expressed as mean ± SD, for normally distributed continuous variables, median (interquartile range) for abnormally distributed variables and number (%) for category variables. ANOVA, or Kruskal–Wallis (KW) test was performed among groups for continuous variables and Chi-square test was used for categorical variables.

Abbreviations:BMI: body mass index; SBP: systolic blood pressure; DBP: diastolic blood pressure; FPG: fasting blood glucose; HbA1c hemoglobin A1c; FCP: Fasting C-Peptide; F-INS: Fasting Insulin; HOMA-IR: Homeostasis Model Assessment of Insulin Resistance; TC: total cholesterol; TG: triglyceride; HDL-C: high-density lipoprotein cholesterol; LDL-C: low-density lipoprotein cholesterol; MGO: methylglyoxal.

### 3.2 Spearman correlation analysis of correlation between FMD and MGO and other indicators

Table 2 presents the relationship between FMD and MGO, along with other clinical indicators. Spearman’s correlation analysis revealed a significant negative relationship between FMD and MGO. (R = −0.611, *p* < 0.001). Additionally, endothelial function was positively correlated with gender (R = 0.192, *p* = 0.002), TC (R = 0.223, *p* < 0.001) and LDL-C (R = 0. 221, *p* < 0.001); And there was a negative correlation with age (R = −0.266, *p* < 0.001) and course of disease (R = −0.191, *p* = 0.002). We found that the indicators MGO (r′ = −0.36, *p* < 0.001) still remained the negative association with FMD afer controlling the variables sex and age, but the correlation coefficient between MGO and FMD decreased. Nevertheless, the correlation between endothelial function and course of disease, TC and LDL-C disappeared following the two variables fixed, and there was a negative correlation between endothelial function.

**TABLE 2:** Correlation FMD with other indicators.

Variables	Spearman’s correlation		Partial correlation	
	*r*	*p* value	*R*	*p* value
Sex (male)	0.192	0.002	-	-
Age (years)	−0.266	<0.001	-	-
BMI (kg/m^2^)	−0.90	0.154	−0.131	0.039
Disease course (years)	−0.191	0.002	−0.117	0.067
SBP (mmHg)	−0.051	0.426	−0.023	0.720
DBP (mmHg)	0.024	0.707	−0.015	0.809
Smoking (%)	−0.049	0.437	0.018	0.779
Drinking (%)	−0.068	0.282	0.009	0.887
HbA1c (%)	0.078	0.222	0.085	0.182
HOMA-IR	−0.009	0.881	−0.141	0.026
TC (mmol/L)	0.223	<0.001	0.083	0.193
TG (mmol/L)	−0.050	0.435	−0.095	0.135
HDL-C (mmol/L)	0.111	0.079	0.036	0.574
LDL-C (mmol/L)	0.221	<0.001	0.093	0.143
MGO (pg/mL)	−0.611	<0.001	−0.36	<0.001

Spearman’s rank correlation coefficient were used to evaluate the unadjusted associations between variables.Partial correlation analysis was further performed to assess the relationship between variables after adjusting for sex and age. *P* < 0.05 was considered statistically significant. Abbreviations:BMI: body mass index; SBP: systolic blood pressure; DBP: diastolic blood pressure; HbA1c hemoglobin A1c; HOMA-IR: Homeostasis Model Assessment of Insulin Resistance; TC: total cholesterol; TG: triglyceride; HDL-C: high-density lipoprotein cholesterol; LDL-C: low-density lipoprotein cholesterol; MGO: methylglyoxal.

### 3.3 Logistic regression analysis of influencing factors of endothelial dysfunction in patients with type 2 diabetes mellitus

Following analysis, MGO was identified as an independent risk factor for endothelial dysfunction in individuals with T2DM. Table 3 demonstrates a significant negative association of endothelial function with per 10% increase in MGO concentration in the univariable analysis (Model 1) (OR 1.099, 95% CI 1.06–1.14). This association remained significant after adjusting for sex, age, BMI, course of disease, hypertension, smoking, and alcohol consumption (OR 1.096, 95% CI 1.05–1.14), as well as further adjustment for HbA1c, HOMA-IR, and TG (OR 1.096, 95% CI 1.05–1.14). In Model 1, the risk of endothelial dysfunction peaked in the Tertile two of MGO levels (ORs = 4.57, 95% CI, 2.82–7.40) compared with the Tertile 1, the odds ratio slightly attenuated in the Tertile 3 (ORs = 3.18, 95% CI, 1.63–6.21). In Model 2, the adjusted ORs were 4.44 (95% CI: 2.68–7.35) for the second tertile and 3.28 (95% CI: 1.64–6.55) for the third tertile (p for trend<0.01)). These associations persisted in Model 3, with corresponding ORs of 4.41 (95% CI: 2.65–7.32) and 3.22 (95% CI: 1.60–6.50) (p for trend<0.01). In model 1, a one standard deviation (SD) rise in MGO (PG/mL) corresponded to a 2.67 -fold higher risk of endothelial dysfunction (95% CI 1.78–4.01). After controlling for sex, age, BMI, disease duration, hypertension, smoking status, alcohol consumption, HbA1c, HOMA-IR, and triglyceride levels, the association remained significant and materially unchanged, with odds ratios of 2.60 (95% CI: 1.70–3.97) in Model two and 2.61 (95% CI: 1.69–4.02) in Model 3.

**TABLE 3:** Association of MGO levels with endothelial dysfunction.

​	Model 1		Model 2		Model 3	
	Or (95%CI)	P Value	Or (95%CI)	P Value	Or (95%CI)	P Value
MGO per 10 pg/mL	1.099 (1.06,1.14)	<0.001	1.096 (1.05,1.14)	<0.001	1.096 (1.05,1.14)	<0.001
MGO tertiles						
Tertile 1	1 (REF)	—	1 (REF)	—	1 (REF)	—
Tertile 2	4.57 (2.82–7.40)	<0.001	4.44 (2.68–7.35)	<0.001	4.41 (2.65–7.32)	<0.001
Tertile 3	3.18 (1.63–6.21)	<0.005	3.28 (1.64–6.55)	<0.005	3.22 (1.60–2.50)	<0.005
*p* Trend	37.67 (8.68–163)	<0.001	32.95 (7.41–146)	<0.001	32.66 (7.34–145)	<0.001
per SD	2.67 (1.78–4.01)	<0.001	2.60 (1.70–3.97)	<0.001	2.61 (1.69–4.02)	<0.001

Logistic regression analysis for the risk of endothelial dysfunction to MGO, tertile.*P* < 0.05 considered statistically significant.Model 1: unadjusted; Model 2: adjusted for sex, age, BMI, disease course, hypertension, smoking, and drinking; Model 3: further adjusted for HbA1c, HOMA-IR, and TG, in addition to confounders in model 2; OR: odds ratio; 95% CI: 95% confidence interval.

**FIGURE 1:**
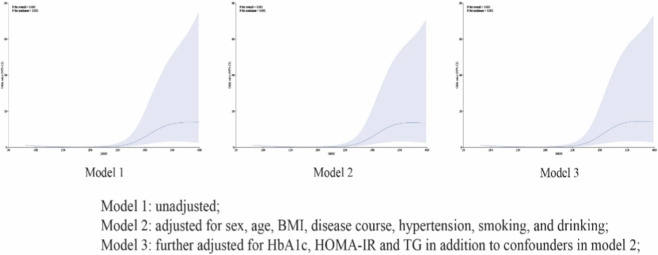
Restricted Cubic Spline (RCS): Association of MGO levels with the risk of endothelial dysfunction. Notes: The dose-response relationship was characterized using restricted cubic spline. *P*_non-linearity_<0.001 indicates a significant deviation from linearity. Model 1: unadjusted; Model 2: adjusted for sex, age, BMI, disease course, hypertension, smoking, and drinking; Model 3: further adjusted for HbA1c, HOMA-IR and TG in addition to confounders in model 2; OR: odds ratio; 95% CI: 95% confidence interval.

To further investigate the dose-response relationships, restricted cubic spline (RCS) analyses were performed to assess the continuous associations between MGO levels and endothelial dysfunction in patients with type 2 diabetes. The restricted cubic spline (RCS) analysis demonstrated a significant relationship between MGO levels and endothelial dysfunction in patients with type 2 diabetes (*P*_overall_<0.001). The risk of endothelial dysfunction increased with rising MGO levels and reached a plateau beyond the second tertile (*P*_non-linearity_<0.001) (Figure 1). And Similar results were obtained when adjusting for gender, age, BMI, course of disease, hypertension, smoking and alcohol consumption (model 2), and HbA1c, HOMA-IR, C-reactive protein, and TG (model 3).

**FIGURE 2:**
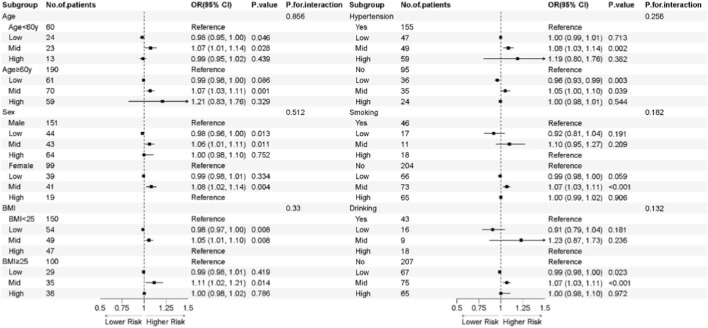
Subgroup analysis for the risk of endothelial dysfunction. Notes: The forest plot displays the odds ratios (OR) and 95% confidence intervals for the risk of endothelial dysfunction across various subgroups. The squareas represent the point estimates of the OR, and the horizontal lines indicate the 95% CI. *P*_interaction_ values were calculated to evaluate the consistency of the association across different sstrata. OR: odds ratio; 95% CI: 95% confidence interval.

Subgroup analyses were performed using Model one to evaluate potential effect modifications by various demographic and clinical characteristics. These findings are visually summarized in the forest plot (Figure 2), with subgroups stratified by age, sex, BMI category, hypertension status, smoking status, and alcohol consumption. The stability of the main association was further supported by consistent effect sizes observed across all predefined subgroups, with no statistically significant interactions observed (all P-interaction >0.05). These findings provide additional evidence supporting the negative association between MGO levels and risk of endothelial function.

### 3.4 Receiver operating curve for MGO predicting endothelial dysfunction

**FIGURE 3:**
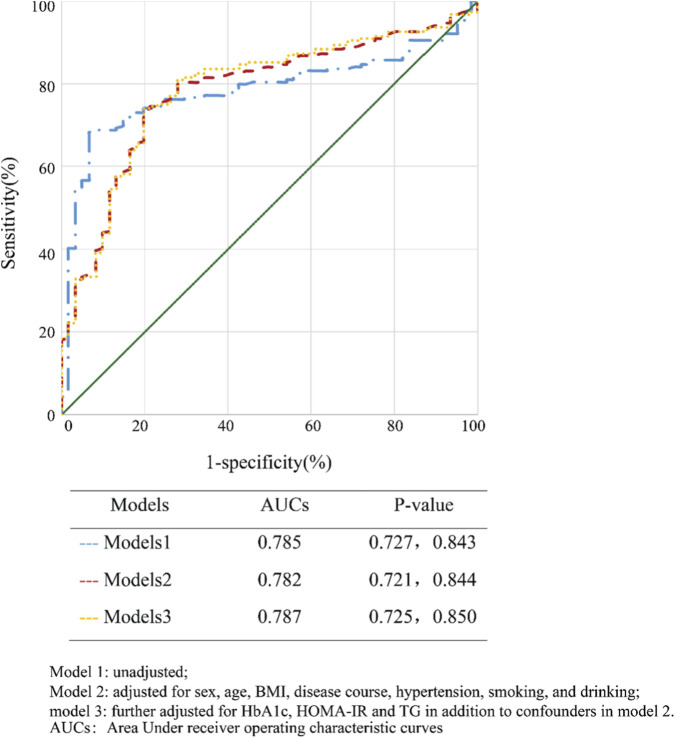
Receiver operating characteristic (ROC) curves for MGO Predicting Endothelial Dysfunction. Notes: Model 1: unadjusted; Model 2: adjusted for sex, age, BMI, disease course, hypertension, smoking, and drinking; Model 3: further adjusted for HbA1c, HOMA-IR and TG in addition to confounders in model 2; AUCs: Area Under receiver operating characteristic curves.

Based on the regression models, ROC curves were constructed to evaluate the predictive performance of MGO for endothelial dysfunction in patients with type 2 diabetes (Figure 3). In the fully adjusted model (Model 3), which included sex, age, BMI, disease duration, hypertension, smoking status, alcohol consumption, HbA1c, HOMA-IR, and triglyceride levels, the area under the receiver operating characteristic curve (AUC) was significantly greater compared to both Model 2 (0.787 vs. 0.782; *p* < 0.001) and Model 1 (0.787 vs. 0.785; *p* < 0.001). Furthermore, the AUC of Model one was also significantly higher than that of Model 2, which was further adjusted for gender, age, BMI, disease duration, hypertension, smoking, and alcohol consumption.

## 4 Discussion

The latest IDF shows that as of 2024, China is the country with the largest number of diabetes patients aged 20-79, up to 148 million, and is expected to continue to grow to 168.3 million by 2050. The total expenditure related to diabetes is 168.9 billion US dollars, accounting for 16.6% of the global expenditure, which poses a great challenge to the medical system and medical consumption. The prevalence of diabetes mellitus is increasing year by year, and the risk of cardiovascular and cerebrovascular diseases in patients with type 2 diabetes mellitus is 72% higher than that in patients without diabetes mellitus, which aggravates the occurrence of macrovascular and microvascular diseases and leads to the rising mortality of diabetes mellitus and its complications (Tufail et al., 2025). Vascular endothelial cells play an important role in regulating vasoconstriction, vasodilation and local inflammation. Chronic hyperglycemia can directly damage endothelial cells and lead to endothelial dysfunction. Compared with glucose, MGO has higher glycosylation reactivity, which can more rapidly produce glycosyl compounds with extracellular proteins, lipids and DNA, form AGEs and reduce the production of nitric oxide. Induces inflammation and oxidative stress, resulting in vasoconstriction and abnormal cell growth, which in turn leads to endothelial dysfunction (Tufail et al., 2025). Endothelial dysfunction is a key factor in the initiation and promotion of diabetic angiopathy, and it is also one of the foundations of diabetic angiopathy. Therefore, it is very important to study endothelial function for the maintenance of cardiovascular homeostasis and the prevention and treatment of diabetic angiopathy. As a new technique for detecting endothelial function based on flow-mediated vasodilation (FMD) of brachial artery, it has the characteristics of easy operation, high accuracy, non-invasive and wide application. Related studies have shown that FMD can be used as an index to evaluate the functional status of vascular endothelium.

Studies have shown that patients with type 2 diabetes have higher plasma levels of MGO, which leads to an increased risk of cardiovascular events and mortality (McTiernan et al., 2019). In this study, the serum MGO level in type 2 diabetic patients with vascular endothelial dysfunction was higher than that in patients without endothelial dysfunction. In addition, patients in the endothelial dysfunction group were older than those in the non-endothelial dysfunction group. Age is an independent risk factor for the progressive decline of endothelial function. The decline of endothelial function with age is caused by the increase of apoptosis and the decrease of regeneration ability of endothelial cells during aging (Ahmed et al., 2024; Tomiyama and Yamashina, 2014).

Our study demonstrated that MGO levels were significantly elevated in patients with endothelial dysfunction compared to those with normal endothelial function. This finding was further supported by a robust inverse correlation between MGO concentrations and endothelial function (measured by FMD), suggesting a direct detrimental role of MGO in vascular homeostasis. In the multivariable logistic regression analysis, MGO emerged as an independent risk factor for endothelial dysfunction, even after adjusting for traditional cardiovascular confounders. For every 10% increase in MGO, the risk of endothelial dysfunction in type 2 diabetes increased by 9.9%, and for every standard deviation increase in MGO, the risk of endothelial dysfunction increased by 2.67 times.

Intriguingly, the risk of endothelial dysfunction did not increase in a strictly linear fashion. We observed that the odds ratio for the second tertile (T2) was higher than that for the third tertile (T3). This non-monotonic trend was fully validated by the restricted cubic spline (RCS) analysis, which illustrated a non-linear dose-response relationship characterized by a plateau effect beyond the T2 threshold. Such a pattern suggests a potential ‘saturation effect,' wherein the pathological impact of MGO on the vascular endothelium may reach a maximal threshold at moderate-to-high concentrations, after which further elevation in MGO does not lead to a proportional increase in risk. Furthermore, the results of our subgroup analysis, visualized through forest plots, confirmed that this negative association remained consistent across diverse clinical strata. These findings collectively underscore the robust and independent role of MGO as a critical mediator of endothelial impairment.

Endothelial dysfunction is caused by many factors, among which diabetes and obesity play a leading role. In patients with dyslipidemia, high low-density lipoprotein cholesterol in the blood is prone to oxidation reaction, forming ox-LDL, which can directly damage endothelial cells, make cells produce excessive reactive oxygen species, further damage the structure of endothelial cells, and impair their function (Kweon et al., 2025; Suraj-Prażmowska et al., 2025). In people with overweight BMI, visceral fat can release free fatty acids and proinflammatory factors to directly damage endothelial cells, or induce excessive production of mitochondrial ROS. Superanions combine with NO to produce peroxynitrite, thus reducing the bioavailability of NO (Rivera et al., 2025). Insulin is an anabolic hormone and a potent vasoactive hormone that regulates vascular tone and tissue perfusion, and insulin resistance is a key factor in the development of type 2 diabetes and its associated cardiovascular complications (Gomez Toledo et al., 2025; Matsuzawa and Lerman, 2014). MGO directly impairs the glucose uptake of islet beta cells, reduces insulin clearance, produces insulin glycosylation, or increases the proliferation and expansion of adipose tissue, reduces capillarization, and eventually leads to insulin resistance. Insulin resistance reduces NO-dependent vasodilation mediated by insulin in arteries and arterioles, increases vascular tension, reduces tissue perfusion, accelerates the formation of atherosclerosis, impairs endothelial structure and function, and eventually leads to endothelial dysfunction (Penna and Pagliaro, 2025; Horton et al., 2025).

This study has limitations. First, this study is a single-center cross-sectional study with a small sample size. Second, Information regarding antidiabetic, antihypertensive, lipid-lowering, or antioxidant medications with an effect on vascular endothelial function, such as GLP-1, SGLT-2, ACEI, and CCB, were uanavailable. Thirdly, serum factors that reflect endothelial function, such as nitric oxide, endothelin-1, and endothelin-nitric oxide synthase, were not examined in this study. Fourthly, in the present study, plasma MGO levels were quantified using ELISA. While this method is more widely accessible and prevalent in clinical settings, we acknowledge that Liquid Chromatography-Mass Spectrometry (LC-MS) remains the ‘gold standard' for MGO measurement, as highlighted by Hanssen (Hanssen et al., 2018). Consequently, we aim to adopt this more precise methodology in our prospective investigations to further validate the current findings.

## 5 Conclusion

To sum up, this study shows that there is a correlation between elevated serum MGO levels and endothelial function (measured by FMD), which can be used as a new indicator to evaluate endothelial function in patients with type 2 diabetes mellitus, and provide a new basis for clinical prevention of diabetic angiopathy.

## Data Availability

The original data used in this study are available from the corresponding authors upon request.
